# Synergistic Antivascular and Antitumor Efficacy with Combined Cediranib and SC6889 in Intracranial Mouse Glioma

**DOI:** 10.1371/journal.pone.0144488

**Published:** 2015-12-08

**Authors:** Merryl R. Lobo, Ayaka Kukino, Huong Tran, Matthias C. Schabel, Charles S. Springer, G. Yancey Gillespie, Marjorie R. Grafe, Randall L. Woltjer, Martin M. Pike

**Affiliations:** 1 Advanced Imaging Research Center, Oregon Health and Science University, Portland, Oregon, United States of America; 2 Department of Biomedical Engineering, Oregon Health and Science University, Portland, Oregon, United States of America; 3 Department of Pathology, Oregon Health and Science University, Portland, Oregon, United States of America; 4 Department of Surgery, University of Alabama at Birmingham, Birmingham, Alabama, United States of America; University of Quebec at Trois-Rivieres, CANADA

## Abstract

Prognosis remains extremely poor for malignant glioma. Targeted therapeutic approaches, including single agent anti-angiogenic and proteasome inhibition strategies, have not resulted in sustained anti-glioma clinical efficacy. We tested the anti-glioma efficacy of the anti-angiogenic receptor tyrosine kinase inhibitor cediranib and the novel proteasome inhibitor SC68896, in combination and as single agents. To assess anti-angiogenic effects and evaluate efficacy we employed 4C8 intracranial mouse glioma and a dual-bolus perfusion MRI approach to measure K^trans^, relative cerebral blood flow and volume (rCBF, rCBV), and relative mean transit time (rMTT) in combination with anatomical MRI measurements of tumor growth. While single agent cediranib or SC68896 treatment did not alter tumor growth or survival, combined cediranib/SC68896 significantly delayed tumor growth and increased median survival by 2-fold, compared to untreated. This was accompanied by substantially increased tumor necrosis in the cediranib/SC68896 group (p<0.01), not observed with single agent treatments. Mean vessel density was significantly lower, and mean vessel lumen area was significantly higher, for the combined cediranib/SC68896 group versus untreated. Consistent with our previous findings, cediranib alone did not significantly alter mean tumor rCBF, rCBV, rMTT, or K^trans^. In contrast, SC68896 reduced rCBF in comparison to untreated, but without concomitant reductions in rCBV, rMTT, or K^trans^. Importantly, combined cediranib/SC68896 substantially reduced rCBF, rCBV. rMTT, and K^trans^. A novel analysis of K^trans^/rCBV suggests that changes in K^trans^ with time and/or treatment are related to altered total vascular surface area. The data suggest that combined cediranib/SC68896 induced potent anti-angiogenic effects, resulting in increased vascular efficiency and reduced extravasation, consistent with a process of vascular normalization. The study represents the first demonstration that the combination of cediranib with a proteasome inhibitor substantially increases the anti-angiogenic efficacy produced from either agent alone, and synergistically slows glioma tumor growth and extends survival, suggesting a promising treatment which warrants further investigation.

## Introduction

The most lethal primary brain tumors are malignant gliomas. The most common glioma, glioblastoma (World Health Organization [WHO] grade IV) is an aggressive and robustly angiogenic tumor associated with a median survival of only 12–16 months despite improved treatments and surgical approaches.[1–3] The limited efficacy of conventional chemotherapeutic agents underscores an urgent need for new therapeutic strategies. While molecularly targeted approaches have been intensively researched in recent years, success is often limited by the redundancy of cellular signaling and the activation of drug resistance mechanisms.[4, 5] Resistance could potentially be circumvented by employing combinations of molecular targets. The anti-angiogenic receptor tyrosine kinase (RTK) inhibitor cediranib targets vascular endothelial growth factor (VEGF), platelet derived growth factor (PDGF) and stem-cell factor receptor (KIT) signaling and is in multiple clinical trials for malignant glioma.[6, 7] We recently reported that Cediranib can effectively reduce 4C8 glioma cell viability *in vitro*, but it has limited efficacy as a single agent with 4C8 glioma *in vivo* [8, 9] consistent with other preclinical studies [10] and clinical reports which have indicated that anti-angiogenic monotherapy largely fails to induce a durable response with malignant glioma.[5, 11–16] Tumors can develop resistance to angiogenic blockade by activating alternative angiogenic pathways or co-opting existing vessels in conjunction with increased invasion of brain parenchyma.[5, 12] Additionally, the exacerbation of hypoxic stress by anti-angiogenic treatment can activate a number of stress response mechanisms in tumor cells, such as those involving HIF1 transcription factors, which facilitate adaptation to hypoxia.[17–19] The current study tested the hypothesis that the combination of Cediranib with the proteasome inhibitor SC68896 substantially enhances *in vivo* efficacy in 4C8 mouse glioma. Inhibition of the proteasome, a key protein degradation mechanism, is well documented to induce potent anti-angiogenic effects in tumors.[20–30] Proteasome inhibition inhibits NFkB, which leads to reduced VEGF and IL-8 expression, critical mediators of angiogenesis.[26, 31–33] Notably, proteasome inhibition also inhibits HIF1α, which promotes angiogenesis and survival under hypoxic tumor conditions.[17–19, 34–38] Numerous studies have reported that proteasome inhibition also inhibits Akt/mTOR signaling, a signaling pathway which is critically involved in survival, proliferation and angiogenesis.[34, 39–45] Proteasome inhibition attenuates cell cycle progression and also modulates apoptotic regulatory protein levels, thereby shifting regulation of apoptosis towards cell death.[21, 46–50] Proteasome inhibitors such as bortezomib (Velcade), have been shown to have significant clinical efficacy in multiple hematologic malignancies such as multiple myeloma and mantle cell lymphoma, but have shown only limited efficacy in solid tumors, including in glioma.[50–53] However, their unique biological activity profile includes inhibition of key oncogenic signaling mechanisms, and effects on apoptosis, angiogenesis, and proliferation, thus making them good candidates for synergizing with other cancer therapeutics. Various studies have demonstrated potentiation of TRAIL induced apoptosis in various cancer cells via combined proteasome inhibition.[41, 54] To our knowledge, the current study is the first to directly assess the potential enhancement of anti-angiogenic effects on the tumor vasculature, with combined proteasome inhibition and RTK angiogenic blockade. As angiogenesis is a key hallmark of tumor progression in high grade gliomas, it is essential that monitoring changes in the development of neovasculature be incorporated into the assessment of the pathophysiological response to therapy[1, 15] Furthermore, as therapeutic efficacy in glioma is linked to key tumor microenvironment variables such as angiogenesis, drug delivery, the effect of hypoxia on tumor biology, and other critical phenomena, it is important that relevant orthotopic *in vivo* models are employed to investigate it. In the current study we employed the syngeneic intracranial mouse 4C8 glioma model, which employs immunocompetent mice and promotes a normal tumor–host interaction. Like clinical glioma, the 4C8 model is highly vascular and exhibits aggressive tumor growth with development of core necrosis.[8, 9, 55, 56] To obtain a noninvasive *in vivo* assessment of the effects of the combined drug regimen, we employed a comprehensive dynamic magnetic resonance imaging (MRI) technique, which assessed tumor vasculature and growth.[8, 56] Dynamic contrast enhanced (DCE) MRI was used to produce high resolution maps of K^trans^ in order to assess vascular extravasation, a key biomarker of tumor neovasculature. Dynamic susceptibility contrast (DSC) MRI was then implemented to measure relative cerebral blood flow and volume (rCBF, rCBV), and relative mean transit time (rMTT). In combination with immunohistological studies of necrosis and vascularization, the current study revealed a synergistic efficacy of combined cediranib and SC68896 in malignant glioma *in vivo*, resulting in enhanced anti-angiogenic effects and increased survival of treated mice. These findings provide a basis for further studies of this novel therapeutic combination in malignant glioma.

## Materials and Methods

### Ethics Statement

Mouse studies were conducted with the approval of the Oregon Health and Science University Institutional Animal Care and Use Committee (protocol #IS00001409) and under the supervision of the OHSU department of Comparative Medicine. All surgery was performed under anesthesia as previously described, and all efforts were made to minimize suffering.[8] After glioma cell implantation and during treatments mice were monitored daily. If mice develop signs of toxicity, such as significant changes in general appearance, eating and other behavior, and body weight (loss>20% of initial), or if MRI indicated that tumor size approached maximal (see below), then mice were euthanized by bilateral thoracotomy following CO2 or pentobarbital injection. After dosing by gavage, animals were monitored closely for 10 minutes and again at 12–24 hours, and were euthanized if any clinical signs of distress developed. Anesthesia and animal monitoring/maintenance within the MRI system was as previously described.[8]

### Cell Culture and Tumor Inoculation

The 4C8 mouse glioma cells were provided by Prof. G. Yancey Gillespie, University of Alabama at Birmingham. The 4C8 cells were originally obtained from Dr. Charissa Dyer, at the Children’s Hospital of Philadelphia, Philadelphia, Pennsylvania, and are derived from a clone of a MOCH-1 tumor that spontaneously arose within the brain of a B6D2F1 mouse transgenic for myelin basic protein promoter–driven *c-neu*.[55] The 4C8 cells were grown as previously described.[8] Female C57BL/6 × DBA/2 F_1_ hybrid mice (B6D2F1) were purchased from Charles River Laboratories (Wilmington, MA). Brain tumors were induced by the intracerebral injection of ~1 × 10^6^ 4C8 cells, suspended in DMEM/F12 (5–10μl) using a stereotaxic frame as previously described ([56]).

### MRI Procedures

T_2_ weighted and contrast enhanced (Magnevist i.p.) T_1_ weighted MR imaging was used to determine when tumor growth initiated. Pretreatment perfusion MRI studies were performed when tumor volume exceeded approximately 3 mm^3^ which generally occurred 10–14 days after inoculation. Mice were then randomized into treatment groups, and treatment started within 24 hours: 1) untreated, n = 4 (vehicle: 1% Tween in phosphate buffered saline (PBS), oral gavage, daily); 2) cediranib, n = 5 (AZD2171, 6mg/kg in 1% Tween/PBS, daily, oral gavage, Selleck Chemicals LLC Houston, TX); 3) SC68896, n = 4 (150 mg/kg in PBS/DMSO 1:2.5, daily, i.p. injection, 4SG AG, Planegg Martinsried, Germany); and 4) cediranib + SC68896, n = 5 (daily). T_2_ weighted imaging was continued biweekly to assess tumor volume. Perfusion MRI experiments were conducted at 0, 10 and 25 (±2) days from tumor growth initiation. Mice were sacrificed, and their brains harvested for histology, when right brain displacement by the tumor approached maximal, or when mice exhibited neurological/behavioral changes, excessive weight loss or skull deformation. For perfusion MRI, mice were anesthetized and mouse lateral tail veins were cannulated as previously described.[8] MR imaging employed a Bruker-Biospin 11.75T small animal MR system and mouse head (20 mm ID) quadrature RF transceiver coil (M2M Imaging Corp.), with custom animal handling and monitoring systems, as previously described.[8] Imaging protocols and parameters were described in detail previously.[8] Briefly, a coronal multislice T_1_ weighted image set was obtained (Paravision FLASH, 98 μm in-plane resolution, 0.5 mm slice width) and used for positioning of the perfusion MRI slice position, and for matching slice position to those in any previous imaging sessions, using anatomical landmarks. A T_2_ weighted image set (Paravision spin echo RARE, same spatial geometry as for multislice T_1_ weighted), was obtained for tumor volume assessment. DCE-MRI was then implemented, (Paravision FLASH, 1 slice, 1 mm slice thickness, 195 μm in-plane resolution) using a fully relaxed precontrast image, followed by the DCE T_1_ weighted image series at the same image slice position, during which Gd-DTPA was injected (Magnevist, Berlex Inc, i.v., 10X diluted, 3.0 μl/g, 0.15 mmol/kg). At the same image slice position and resolution, DSC-MRI was then implemented, employing the SPIO agent Feridex (Berlex Inc, 4X diluted, 2.4 μl/g, 26.9ug iron/g).

### Image Processing

DCE-MRI parameters were computed voxelwise as previously described ([8]) using the Extended Tofts-Kety model ([57, 58]) with custom pharmacokinetic modeling software, estimating the arterial input function directly from measured tumor curves ([59, 60]). Fully relaxed M_0_ images were used to compute pre-injection longitudinal relaxation time to enable quantitative estimates of the volume transfer constant K^trans^ (min^−1^).([56]) DSC-MRI perfusion parameters were calculated using the Jim software package (Xinapse Systems LTD, Northants, UK) as described in detail previously ([8, 56, 61]) resulting in the generation of parametric maps for cerebral blood flow (CBF in ml blood/100g tissue/ minute), cerebral blood volume (CBV in blood volume percentage of total tissue volume), and mean transit time (MTT in seconds). MR images and maps were analyzed using Jim software as described previously.[56] Tumor volumes and mean parameter values were obtained from the images/maps employing regions of interest drawn at the tumor perimeter, as previously described in Pike *et al* 2009.[56] Tumor volumes were obtained from the T_2_ weighted images. The CBF, CBV and MTT, were reported as relative to contralateral (rCBF, rCBV, rMTT), to reduce measurement error and effects from variations in intracranial pressure, blood pressure, and depth of anesthesia.

### Histology

Immunohistological procedures and analysis were described in detail previously.[8] Briefly, after MRI study termination, mice were euthanized and their brains were stored in 10% formalin for 24–48 hours, embedded in paraffin, and sectioned. Slides were processed and stained with Hemotoxylin (Sigma-Aldrich) and Eosin (Sigma-Aldrich) and analyzed for necrotic regions employing a minimum of 3 tumor samples/treatment group, a minimum of 2 tumor sections per tumor and 3 image fields per section. Mean vessel density (MVD) and mean lumen size were quantified within the tumors from CD31 immunostained sections, employing a minimum of 3 tumors per treatment group, with 2 sections per tumor and 3–6 adjacent 10X fields per section. The number of CD31 stained vessels per field were averaged to obtain MVD. Lumen areas were quantified in vessels with visible lumens in which the entire circumference of the vessel wall was visible, using the Jim software package. To assess apoptosis, cleaved caspase-3 positive cells within viable (non-necrotic) tumor tissue were quantified in cleaved caspase-3 stained immunohistochemical sections (Cell Signaling #9664, rabbit monoclonal, dilution 1:2000). Additional sections were stained for Ki67 (Cell signaling #1222, rabbit monoclonal, mouse specific, dilution 1:400). Both the caspase-3 and Ki67 immunostains were visualized using biotinylated secondary antibodies, avidin-biotin peroxidase complex, and DAB as the chromogen. The percentage of Ki-67 positive nuclei within the tumors was determined using the ImmunoRatio application within Image J.[62] The cleaved caspase-3 and Ki67 analyses both employed 2–5 tumors per treatment group, with 2 sections per tumor and 3–6 adjacent 10X fields per section. With all histological analyses, data from the analyzed visual fields within a given tumor were first averaged to obtain individual tumor averages, which were then employed for statistical analysis and averaged to obtain treatment group means. All of the above histological analyses were implemented under the guidance of experienced neuropathologists (MRG and RLW).

### Statistics

Values are expressed as mean ± SEM. For dynamic MRI parameters (except K^trans^), the two-tailed Student’s t test (Graphpad Prism 6, La Jolla, CA), was employed to test for differences at 0, 10±2 days after tumor growth initiation versus untreated (time points corresponding to ≥3 surviving mice). The one-tailed Students t test was employed in the statistical analysis for mean tumor K^trans^. Differences in median survival were tested using the log-rank test. The two-tailed Students t test with Welch’s correction was employed for the histological analyses.

## Results

**Fig 1:**
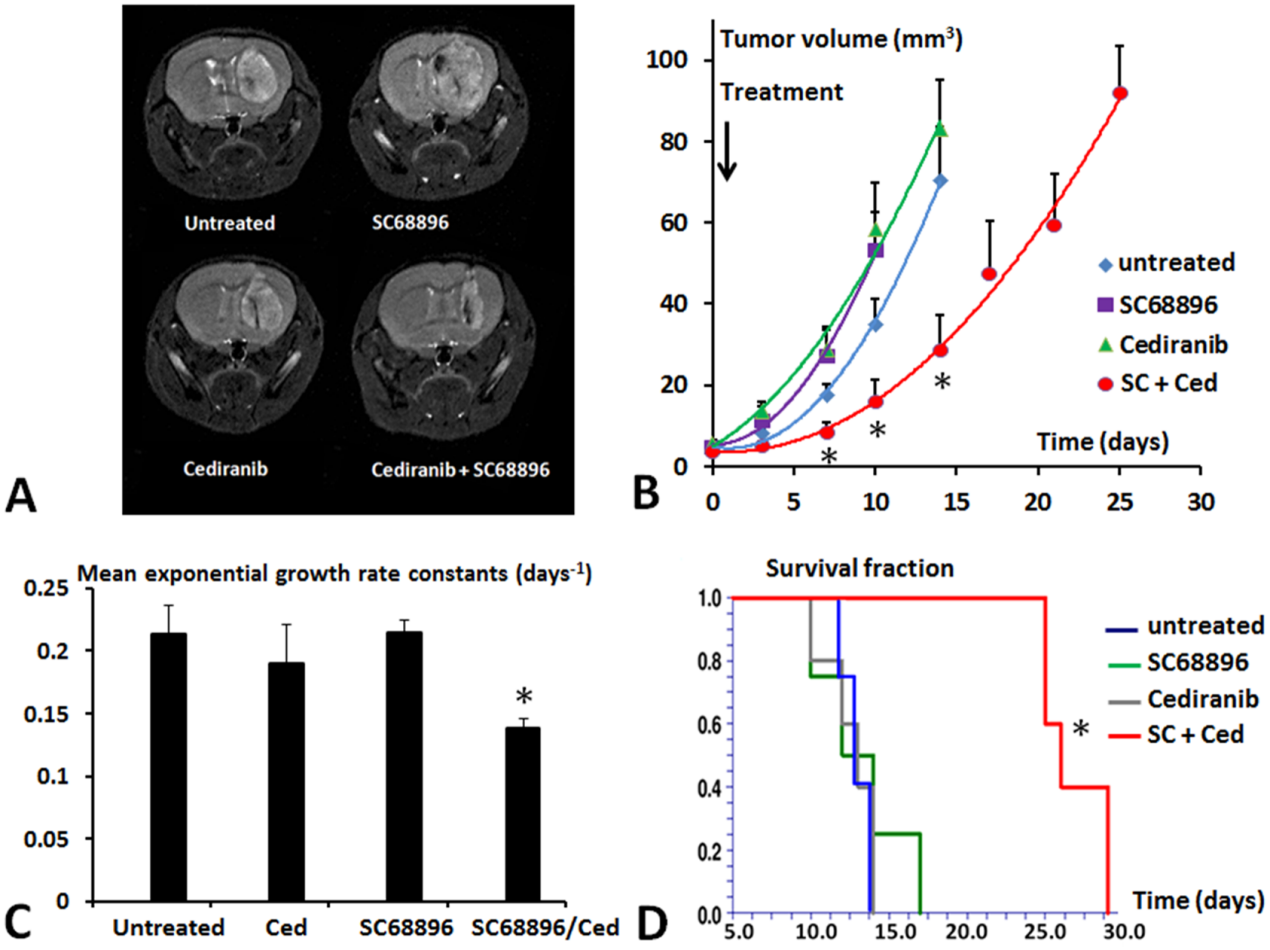
Cediranib/SC68896 treatment slows tumor growth and increases mouse survival. **A)** Representative T_2_ weighted coronal mouse brain MR images obtained at 7 days after tumor growth initiation for the four treatment groups. **B)** Tumor growth curves are shown for the four treatment groups, in tumor volume versus time after tumor growth initiation. Curves are indicated for time points corresponding to ≥3 surviving mice, *: p<0.05 versus untreated. **C)** Mean exponential growth rate constants are shown for the four treatment groups, *: p<0.05 versus untreated. **D)** Kaplan-Meier survival curves for the four treatment groups (in days from tumor growth initiation), *: p<0.01 versus untreated.

Fig 1A shows representative coronal T_2_ weighted mouse head MR images obtained 7 days after tumor growth initiation for the various treatment groups. The images clearly delineate the tumors in the mouse brains, and indicate a substantially smaller tumor size with combined cediranib/SC68896 treatment compared to single agent or vehicle treatment. Tumor growth curves obtained from multislice T_2_ weighted images are shown in Fig 1B, with tumor volume plotted against time from tumor growth initiation, and indicate rapidly growing tumors in vehicle-treated mice. Mouse treatment began on day 1, and notably cediranib or SC68896 single agent treatment had no effect on tumor growth. In contrast, tumors in mice treated with the cediranib/SC68896 combination grew at a significantly lower rate, with 60% smaller tumors than vehicle-treated at 10 days. Confirming this are the mean exponential growth rate constants obtained from exponential fits to individual tumor growth curves shown in Fig 1C, indicating a significantly lower growth rate (in days^−1^) for cediranib/SC68896 (0.14±0.007) versus vehicle-treated (0.21±0.02, p<0.01)), with cediranib (0.19±0.03) and SC68896 (0.21±0.01) showing values similar to untreated. Kaplan-Meier survival curves in Fig 1D show that mouse survival was substantially extended in the cediranib/SC68896 combination group in comparison with other treatment groups, with a median survival (days) of 26±0.91 (p<0.01 vs vehicle) compared to mice given cediranib (13±0.75), SC68896 (13±1.49), or vehicle treated (13.5±0.47).

**Fig 2:**
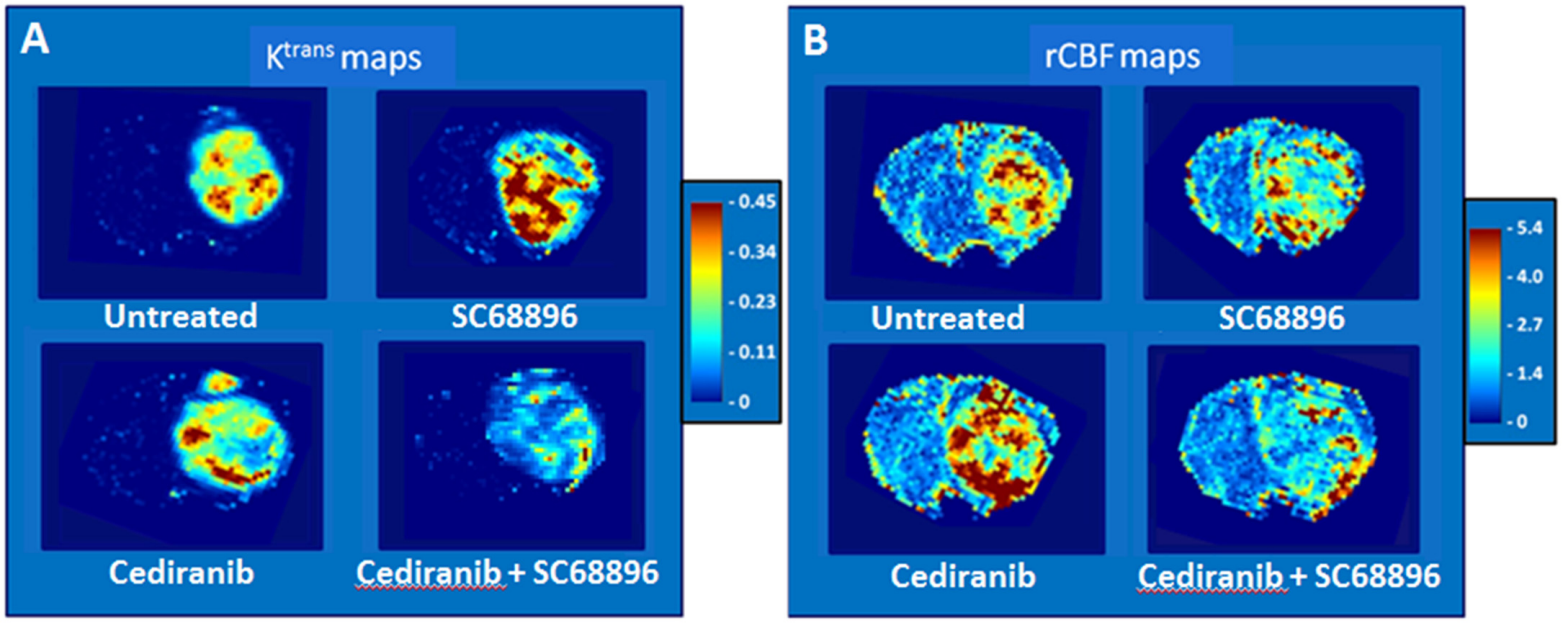
Cediranib/SC68896 treatment reduces tumor K^trans^ and rCBF. **A)** K^trans^ (min^−1^) maps obtained from representative tumors from each treatment group during the final week of MRI evaluation; **B)** rCBF parametric maps (non-brain regions masked) from the same tumors as in A (CBF color scale is relative: contralateral hues set to ~unity).

Fig 2A and 2B show representative K^trans^ and rCBF mouse brain parametric maps using the dual bolus-tracking DCE/DSC perfusion MRI approach, obtained from the 4 treatment groups at the final MRI evaluation time point. In the normal brain tissue, the intact blood brain barrier prevented contrast agent extravasation, resulting in negligibly small K^trans^ values. In contrast, tumor tissue exhibited elevated and heterogeneously distributed K^trans^ values, indicating compromised blood brain barrier integrity. The areas of elevated K^trans^ were particularly prevalent within tumors in SC68896 treated mice. Cediranib treatment generally decreased K^trans^ values in the tumor core, but failed to have an effect along the rim, consistent with previous studies from our laboratory.[8] In contrast, the combined administration of cediranib/SC68896 resulted in substantially lower K^trans^ values. Fig 2B indicates that as with K^trans^, the tumor CBF maps also exhibited elevated and heterogeneously distributed values, in contrast to normal brain. Analogous to what was observed on the K^trans^ maps, the rCBF maps indicated the presence of a resistive angiogenic tumor rim in response to cediranib treatment. In contrast, both single agent SC68896 treatment, and the combined cediranib/SC68896 treatment, decreased overall tumor rCBF, and effectively reduced rCBF in the tumor rim.

**Fig 3:**
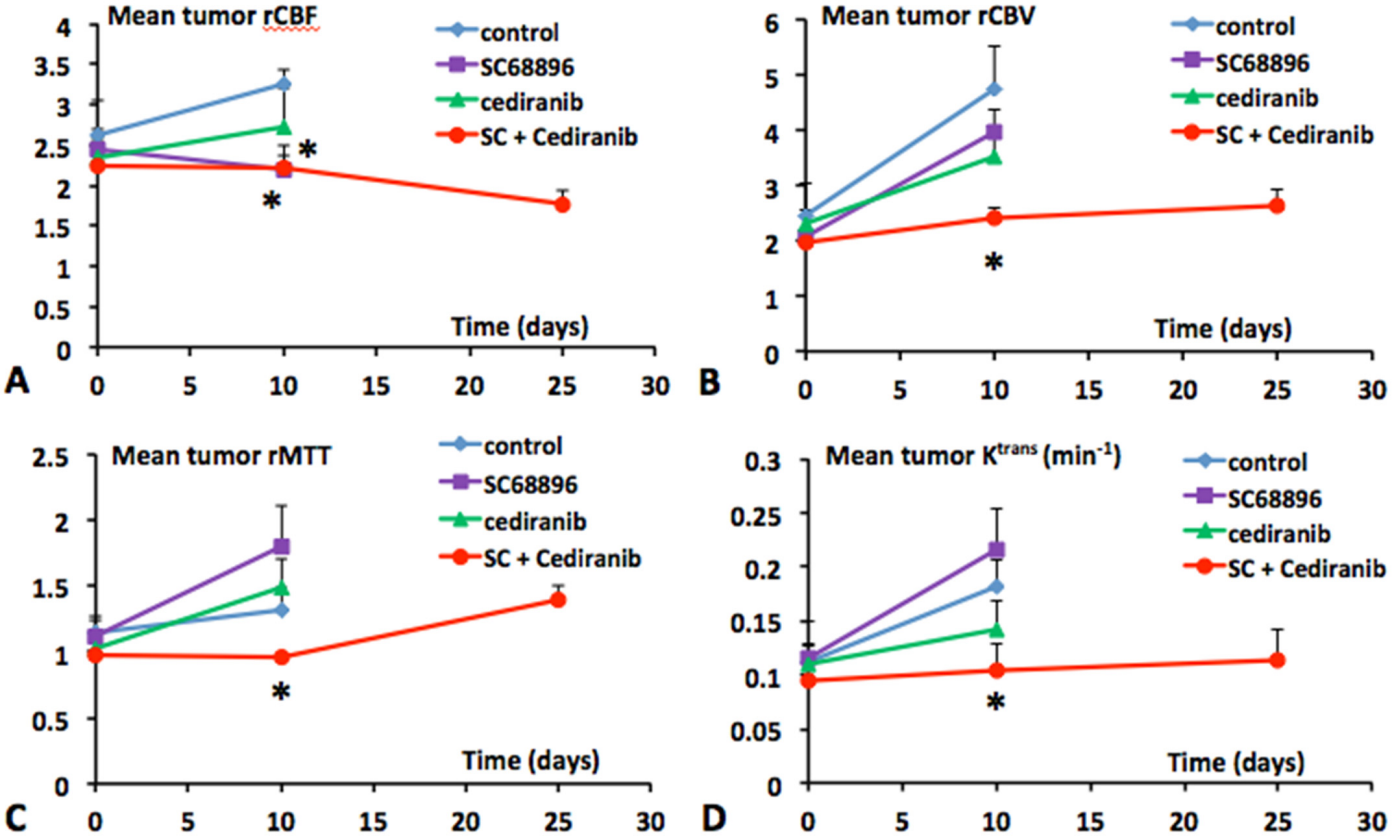
Cediranib/SC68896 treatment prevents increases in mean tumor rCBF, rCBV, rMTT and K^trans^. Mean tumor **A)** rCBF, **B)** rCBV, **C)** rMTT and **D)** K^trans^ (min^−1^) values plotted versus the time from tumor growth initiation for the four different treatment groups (untreated, SC68896, cediranib, cediranib/SC68896). Curves are indicated for time points corresponding to ≥3 surviving mice; *: p<0.05 versus untreated.

**Fig 4:**
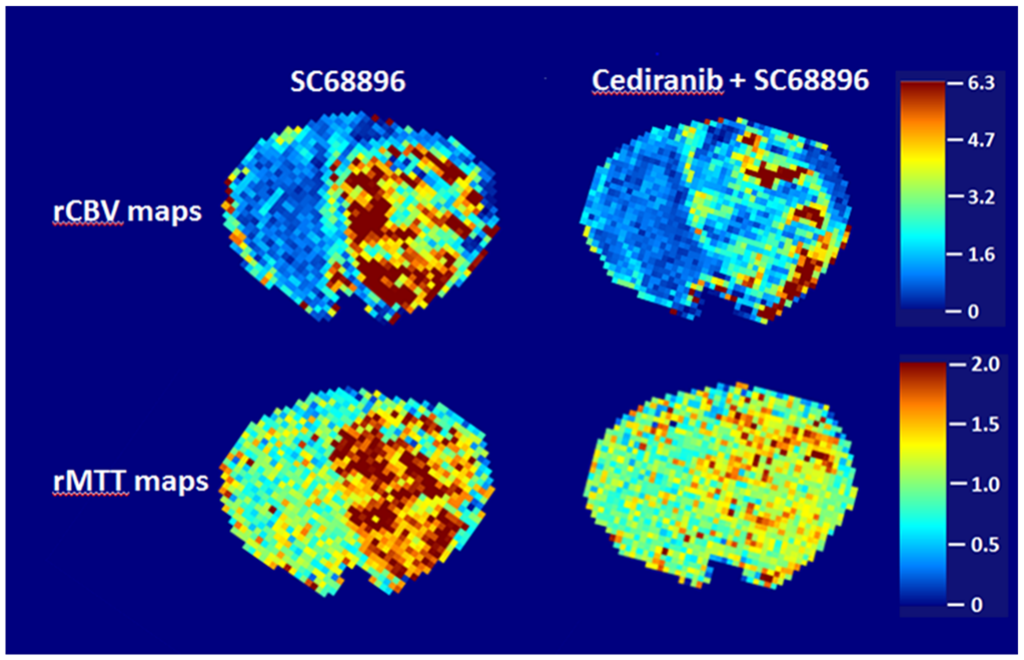
Cediranib/SC68896 treatment reduces tumor rMTT and rCBV in comparison to SC68896 alone. **A)** rCBV and **B)** rMTT parametric maps (non-brain regions masked) obtained from the same tumors depicted in Fig 2, from the SC68896 and cediranib/SC68896 treatment groups during the final week of MRI evaluation (CBF color scale is relative: contralateral hues set to ~unity).

Fig 3 shows the variation of mean tumor rCBF, rCBV, rMTT and K^trans^ with time from the start of tumor growth, for the four treatment groups. On day 0, mean tumor rCBF values of between 2.2–2.6 were observed for the four treatment groups (Fig 3A). The mean tumor rCBF increased substantially with tumor growth in vehicle-treated mice. Fig 3B shows that the rCBV increased faster than rCBF in vehicle-treated tumors, which suggests a progression to a more chaotic and inefficient vascular network. Further supporting this is that rMTT, a parameter inversely related to vascular efficiency (Fig 3C) and is defined by the relation MTT = CBV/CBF, tended to increase with tumor growth in vehicle-treated tumors. Also consistent with a progressive development of abnormal tumor neovasculature is the increasing K^trans^ (Fig 3D) observed in vehicle-treated tumors, indicating increased contrast agent extravasation from the vasculature. Fig 3 shows that cediranib treatment alone did not produce significant changes in mean rCBF, rCBV, or rMTT from those observed in vehicle treated mice, although a tendency for lower rCBF and rCBV values was observed. Consistent with our previous findings, Cediranib also tended to decrease mean tumor K^trans^ in comparison to untreated. Because under most conditions, K^trans^ is proportional to the permeability-surface area product per unit volume of tissue, K^trans^ changes can be indicative of changes in total vessel surface area and/or altered vascular permeability, a property which is independent of vessel surface area. Decreased vascular permeability would be consistent with cediranib’s known inhibition of VEGF receptors. However, a contribution from reduced vessel surface area would be consistent with the observed tendency towards lower rCBV values with cediranib. Consistent with the parametric maps shown in Fig 2, single agent SC68896 treatment significantly decreased rCBF in comparison to vehicle-treated at day 10 of tumor growth/treatment. This occurred without concomitant reductions in rCBV, a trend consistent with an exacerbation of vessel inefficiency, as further suggested by the large increase in rMTT. The concomitant large increase in K^trans^, indicating increased contrast agent leakage across the blood-tumor barrier, lends support to the concept that single agent treatment with SC68896 promotes an abnormal vessel phenotype. In contrast, the combined cediranib/SC68896 treatment effectively restricted increases in mean tumor rCBF, rCBV, and rMTT, resulting in values significantly lower than vehicle treated at day 10. Furthermore, K^trans^ increases were also entirely prevented in SC68896/cediranib treated tumors. The increased survival in the combined treatment group enabled extended perfusion MRI monitoring, which indicated that prevention of rCBF, rCBV and K^trans^ increases continued to day 25 in the combined treatment group. A downward trend was observed with rCBF at that time point, which in combination with the stable rCBV, was consistent with rMTT trending slightly upward. The rCBV and rMTT maps in Fig 4 further exemplify the qualitatively different vascular effects of SC68896 and combined cediranib/SC68896, obtained from the same tumors and treatment stage as those depicted in Fig 2. Relatively high values of rMTT, as well as high rCBV values, were generally observed across the SC68896 treated tumor. In contrast, the rCBV and rMTT values were comparatively attenuated in the cediranib/SC68896 treated tumor. The differences observed between the two groups in rCBV and rMTT occurred despite the generally similar patterns of rCBF in the same tumors (Fig 2). Both SC68896 and combined cediranib/SC69986 reduced rCBF in comparison to the untreated tumor, but SC69986 monotherapy induced this effect without decreasing rCBV, rMTT or K^trans^, consistent with the mean values for the experimental groups shown in Fig 3. The data suggest a maintenance or exacerbation of tumor vasculature inefficiency with SC68896 treatment. In contrast, combined cediranib/SC68896 treatment also reduced flow but at the same time reduced vascular extravasation and improved flow efficiency, possibly suggesting a vascular remodeling process which induces a more normal tumor vascular phenotype.

**Fig 5:**
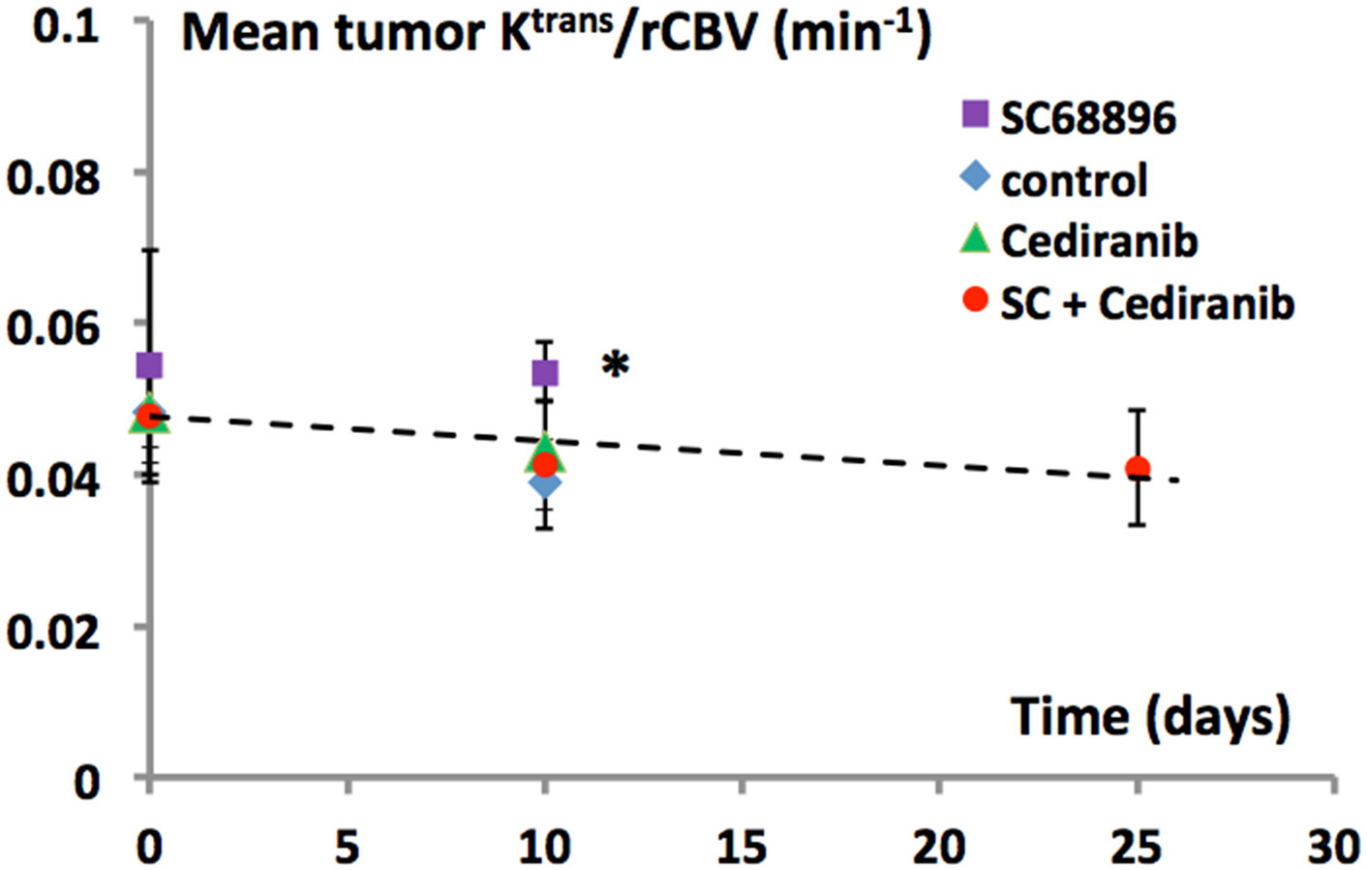
Decreased K^trans^ with cediranib/SC68896 treatment is largely related to decreased rCBV and vessel surface area. The K^trans^/rCBV ratio is plotted versus the time from tumor growth initiation for the different treatment groups. Dashed line was fit to the mean values of the various treatment groups (posttreatment means excluded the SC68896 group). Similar values of K^trans^/rCBV for the untreated, cediranib, and cediranib/SC68896 groups were observed at the 10 day time point. Moderately but significantly higher values were observed in the SC68896 group versus untreated (p<0.05). Within each group, K^trans^/rCBV was relatively stable over time. See text for details.

Because of similarities between the rCBV and K^trans^ timecourses (Fig 3B and 3D) for the various treatment groups, we further analyzed their relationship by plotting the four [K^trans^/rCBV] time-courses in Fig 5. Interestingly, essentially all time-dependence is removed, with that for K^trans^/rCBV indicating a marginally negative slope. For a given time point similar K^trans^/rCBV values were indicated for the experimental groups. While the SC68896 group indicated a significantly higher K^trans^/rCBV than untreated, the magnitude of the difference was small.

A straightforward derivation in the S1 Appendix shows that K^trans^/CBV is proportional to P_CA_/d and thus to k_pe_ [P_CA_ is the capillary wall contrast agent (CA) permeability coefficient, d is the *average* capillary diameter, and k_pe_ is the unidirectional rate constant for CA extravasation]. While, for Gd chelate CA’s, K^trans^ is the P_CA_S product [S1 Appendix; S is the *total* blood vessel surface area], P_CA_ and k_pe_ are pure measures of CA permeability. Thus, the observed lack of variation of K^trans^/rCBV indicates that almost all K^trans^ variation seen in this study is due to variation of S (CBV); *i*.*e*., vascularization changes.

**Fig 6:**
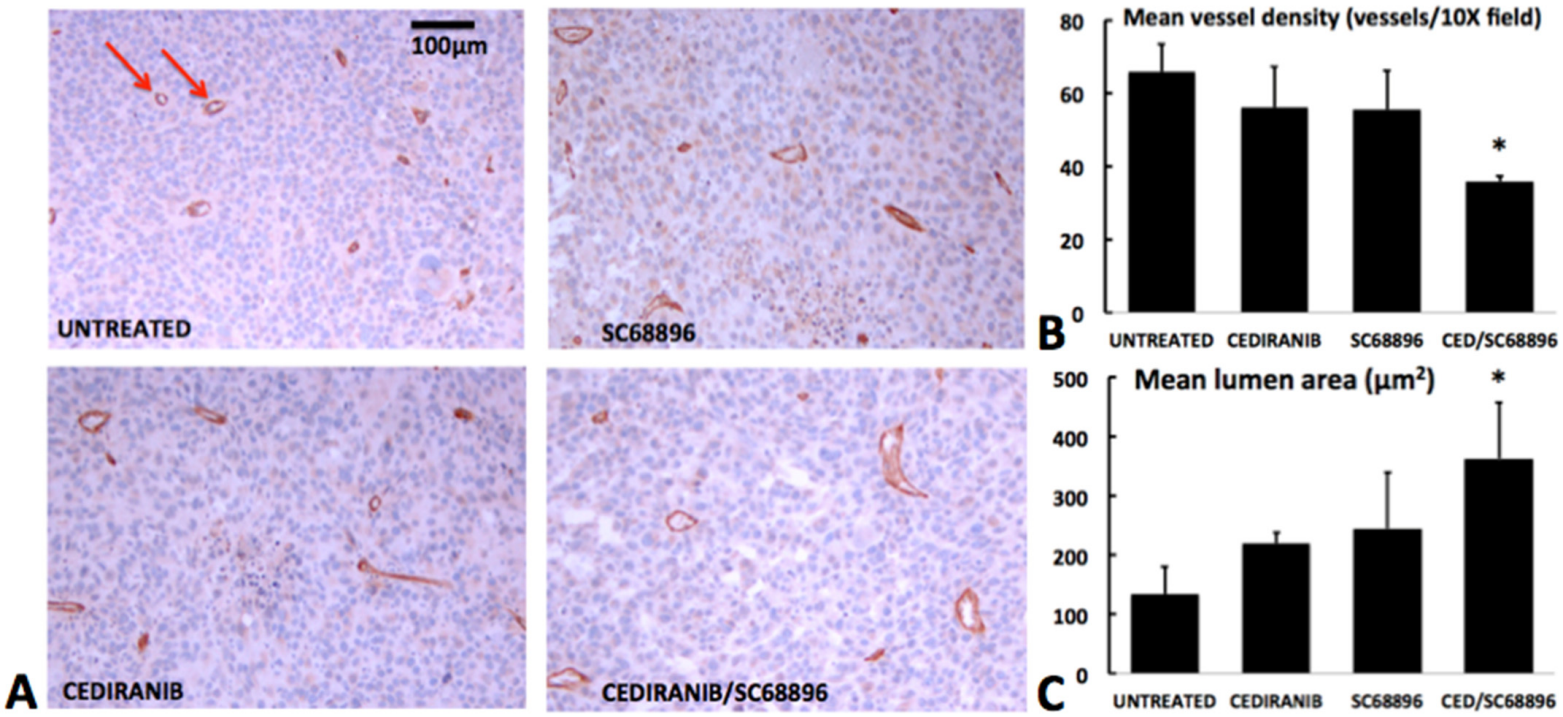
Cediranib/SC68896 treatment reduces tumor mean vessel density and increases mean lumen area. **A)** Representative histological tumor sections with CD31 vascular staining (brown) and hematoxylin nuclear counter stain (blue) from the four treatment groups. Microvessel examples are marked by arrows. **B)** MVD quantified from multiple CD31 stained sections for the four treatment groups, indicating a significantly lower level for cediranib/SC68896 versus untreated (p<0.05). **C)** Mean lumen areas are indicated for the various treatment groups, with that for cediranib/SC68896 indicating a significantly higher level than untreated (p<0.05).

To further define the vascular changes that were induced by the treatments, we examined CD31 stained sections obtained after the completion of the longitudinal MRI studies. Fig 6A indicates typical tumor CD31-stained tumor sections from each of the treatment groups. Mean vessel density (MVD) was determined, and vessel lumen areas were measured from vessels with quantifiable lumens, constituting 28±17% (SD) of vessels observed. Vessel lumens which were too small to be measured, or not completely visible in cross section, were not quantified. Hence the quantified vessels likely only included the larger capillaries, as well as small arteries and veins/venules. Fig 6B indicates a substantially and significantly reduced MVD for the combined cediranib/SC68896 treatment group in comparison to untreated. Fig 6C indicates that the vessel lumen size significantly increased for the cediranib/SC68896 group in comparison to untreated, exhibiting lumen areas almost threefold larger. Collectively, the graphs in Fig 6B and 6C suggest an association between increasing lumen size and MVD reduction.

**Fig 7:**
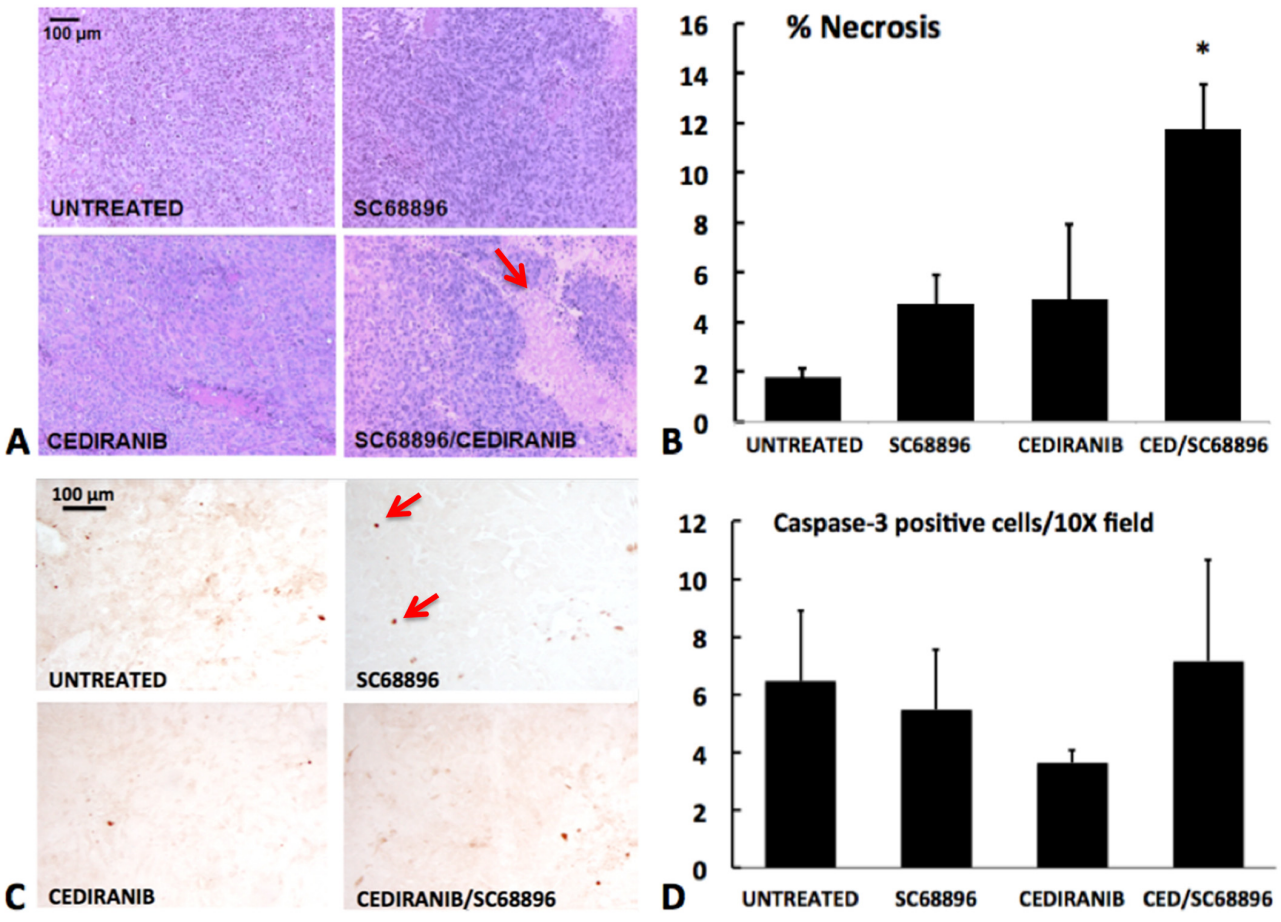
Cediranib/SC68896 treatment increases tumor necrosis. **A)** Representative H&E stained sections from the four treatment groups, indicating areas of necrosis, which appear as loss of nuclear staining in areas of vacuolated tissue (see arrow). **B)** Percent necrosis, quantified from multiple H&E stained sections, for the four treatment groups. Significantly greater necrosis was observed for the cediranib/SC68896 versus untreated (p<0.01). **C)** Viable tissue sections stained for caspase-3, a marker of apoptosis, are shown for the different treatment groups. Arrow indicates caspase-3 positive cell examples. **D)** The mean number of caspase-3 positive cells/10X field in viable tissue is shown. No significant differences were observed among the treatment groups.

**Fig 8:**
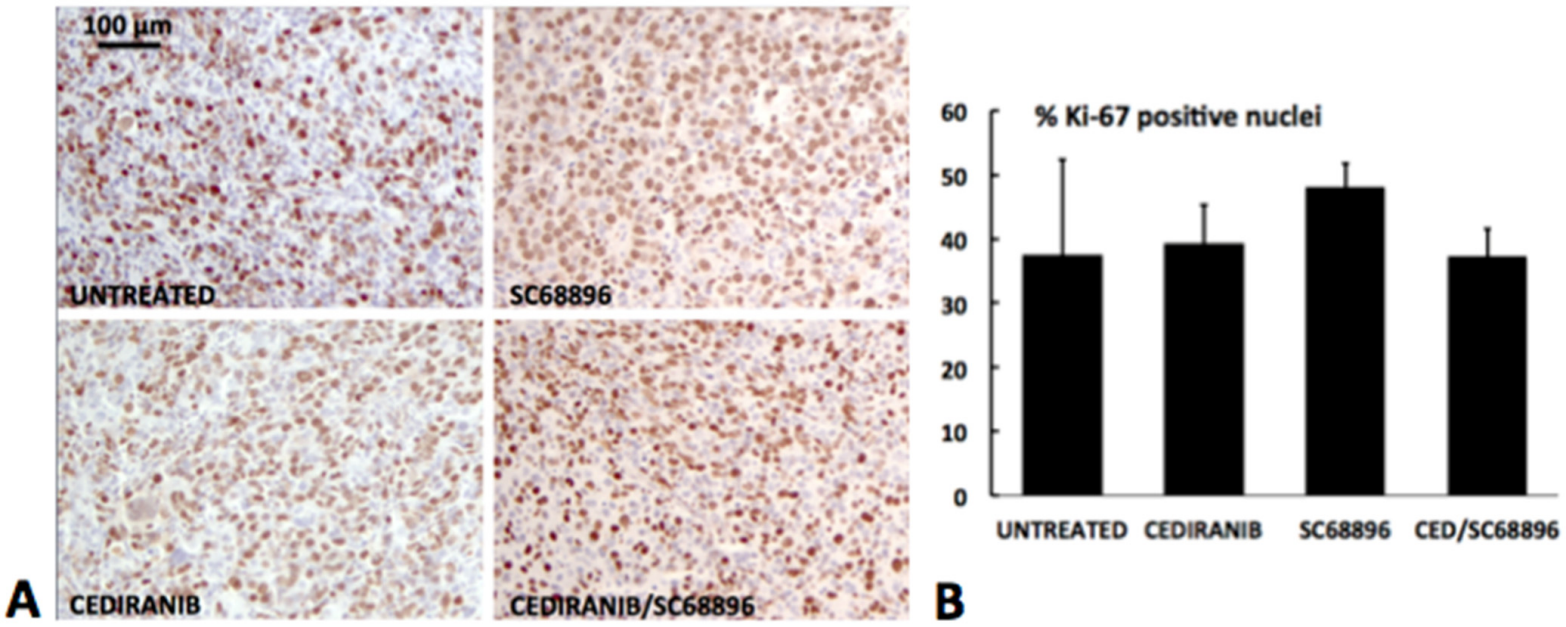
Treatment with Cediranib and/or SC68896 does not reduce cell proliferation. **A)** Representative Ki67 (brown) (with blue nuclear counterstain) are shown for the different treatment groups, indicating a generally high proportion of Ki67 positive nuclei, indicative of robust cell proliferation. **B)** The percentage of Ki67 positive cells is indicated for the various treatment groups. Generally similar values were indicated for the various treatment groups.

To investigate the effect of the treatments on tumor cell death the percent tissue necrosis was quantified from the H&E stained sections, and apoptotic cell death was quantified in viable tissue from cleaved caspase-3 stained sections. Fig 7A and 7B, show a marked increase in percent tissue necrosis with cediranib/SC68896 combination treatment in comparison to all other treatment groups (p<0.01). Fig 7C and 7D indicate that in viable (non-necrotic) regions, relatively few cells stained positively for cleaved caspase-3, and significant differences were not observed between experimental groups. Higher levels were generally observed within necrotic regions but were not quantified to avoid masking effects within viable tissue and because of significant contributions from nonspecific staining. To assess cell proliferation, the percent of cells which stained positively for Ki67 was quantified, as shown in Fig 8. Generally high and similar levels of cell proliferation were observed across the experimental groups, and significant differences were not observed.

## Discussion

The dismal prognosis for malignant glioma necessitates development of new therapeutic strategies. Single agent anti-angiogenic treatment, with either small-molecule or large-molecule therapeutics, has generally been shown to be of limited efficacy in glioma.[5, 11–16] The current study is predicated on the concept that anti-angiogenic and anti-tumor efficacy can be achieved via rational combinations of molecularly-targeted agents. We employed a unique perfusion MRI approach to quantitatively investigate the *in vivo* anti-vascular and anti-tumor effects of the novel cediranib/SC68896 combination using the syngeneic 4C8 intracranial mouse glioma model. SC68896 was employed in the current study due to its documented ability to exert antiglioma activity *in vivo* in a mouse experimental xenograft, in contrast to bortezomib. [49, 53, 63] It has been shown to effectively inhibit the proteasome in glioma cells, inducing accumulation of p21 and p27 proteins, cell cycle arrest, caspase cleavage, and induction of apoptosis. Consistent with our previous findings with cediranib in 4C8 glioma, we found that single agent cediranib did not affect tumor growth or survival, suggesting resistance of intracranial 4C8 glioma to cediranib, modeling what has been observed clinically. Similar to our previous observations, we observed a tendency for cediranib to more effectively target the tumor core vasculature than the tumor rim, which often exhibited a compensatory neovascularization response. However, mean tumor perfusion MRI tumor parameters were not significantly altered from untreated. Similar to cediranib, SC68896 also did not affect 4C8 tumor growth or survival as a single agent, differing from a previous report, which noted a measurable extension of mouse survival with a different glioma model.[49] Our perfusion MRI data however, revealed that SC68896 substantially reduced mean tumor rCBF in comparison to untreated, showing more efficacy in this regard than cediranib. Most interestingly, SC68896 did so without substantially reducing rCBV, suggesting that the reductions in rCBF were accompanied by decreased vascular efficiency, an observation supported by the high levels of mean rMTT that were also observed. High K^trans^ levels were also observed, indicating increased contrast agent extravasation. Although the specific etiology is unknown, these data indicate that SC68896 substantially impacted tumor vascular development, consistent with an exacerbation of vascular abnormality. In contrast to our observations with the single agent treatments, combined cediranib/SC68896 substantially delayed tumor growth, doubled median survival, and greatly increased tumor necrosis, clearly indicating an effective synergy between the agents. The perfusion MRI data also indicated that a strong synergy exists in regards to anti-angiogenic effects. The increases in rCBF, rCBV, rMTT, and K^trans^ with tumor growth that were observed in the untreated and other groups were entirely prevented by the combination, resulting in substantially reduced values of all of these parameters at advanced stage of tumor growth in comparison to untreated. The relatively low rCBV and rMTT values are consistent with an increased vascular efficiency in the combination group in comparison to untreated. As demonstrated in Figs 3 and 4, prominent differences in rCBV and rMTT were also observed between the SC68896 and cediranib/SC68896 groups. Further evidence for vessel remodeling with cediranib/SC68896 was provided by the immunohistochemical analysis, which indicated that the treatment significantly reduced MVD to levels almost half that of untreated, in tandem with a near tripling of mean vessel lumen area. Aspects of the changes in vascular properties that we observed with cediranib/SC68896 treatment are consistent with what is sometimes called a “normalization” of the tumor vascular phenotype, an effect which can which can occur during an early time window of anti-angiogenic treatment. Vascular normalization generally refers to an increased vascular efficiency due to a pruning of chaotic, dilated, and permeable tumor vasculature in favor of more organized, homogeneous, and efficient vasculature.[10, 12, 64, 65] While preclinical and clinical evidence for this effect has accumulated, its extent and the time window over which it occurs is highly variable and model dependent, with a notably short time window in orthotopic rodent models.[10, 64, 65] Improved vascular efficiency is not synonymous with increased blood flow, and there are relatively few reports of improved CBF during a normalization window.[66] A salient feature of vascular normalization, in addition to increased vascular efficiency, is decreased vascular extravasation leading to a reduction of interstitial fluid pressure, which purportedly improves vascular efficiency and delivery of oxygen, nutrients and drugs. The marked reduction in K^trans^ that we observed with the combined cediranib/SC68896 treatment, indicates reduced vascular extravasation which may contribute to increased vascular efficiency as indicated by the reduced rMTT. Conversely SC68896 treatment induced an elevated K^trans^ concomitant with elevated rMTT and low rCBF, possibly resulting from increased vascular extravasation and interstitial fluid pressure. Consistent with the normalization concept, cediranib/SC68896 improves vascular efficiency, reduces vascular extravasation, and also prunes the vasculature as indicated by the reduced MVD and rCBV. Our study shows that the restriction of rCBF, rCBV and K^trans^ increases continues to day 25 in the combined treatment group. A tendency for decreased rCBF was observed at day 25, which in combination with an unchanged rCBV, resulted in a moderately increased in rMTT. Because neither rCBF or rCBV increased, this does not represent a developing resistance to anti-angiogenic effects, but instead suggests that while the optimum vascular efficiency observed during the combined treatment period was at day 10, the vascular remodeling process continued to the late tumor stage, limiting the increases in vascular extravasation, flow and volume. Our observation of increased lumen size in the combined cediranib/SC68896 treatment group is not typically reported in connection with normalization, which generally is associated with reduced vessel diameter. While this aspect may differ from the classic description of vascular normalization, it is not unexpected. A pruning of excess and inefficient vasculature might be expected to increase flow and lumen diameter in the remaining larger blood vessels, those which were primarily assessed by the lumen analysis. The observation that cediranib/SC68896 treatment uniquely reduced the panel of MRI parameters in Fig 3 and was the only treatment to significantly alter MVD and lumen size further supports the concept that they are interconnected and represent different aspects of a vascular remodeling process.

Our study employs a novel strategy to distinguish the extent of vascular extravasation from permeability itself. While the term permeability is often employed broadly, *ie* a measure of extravasation from the vasculature, we use it in the strictest sense [the probability for CA capillary escape per unit time, independent of the total capillary surface area, S]. Permeability is measured by P_CA_ or k_pe_. On the other hand, K^trans^ is the P_CA_S product.[67] Hence, vascular extravasation and permeability represent different quantities, with the former being dependent on permeability but also on the extent of surface area. Our comprehensive dual-bolus DCE/DSC MRI approach enabled calculation of the K^trans^/rCBV ratio (S1 Appendix), which is proportional to P_CA_ (the permeability coefficient) and also to k_pe_, the (unidirectional rate constant for CA extravasation). Because K^trans^/rCBV is relatively stable over time and exhibits largely similar values for the experimental groups (Fig 5), it suggests that P_CA_ and k_pe_ changes play a relatively minor role in the evolution of different K^trans^ values with tumor progression, and under the various treatment conditions. It should be noted that average capillary diameter (d) factors into the relationship between K^trans^/CBV and P_CA_ (S1 Appendix). We determined that the lumen area of the larger vessels differed between certain treatment groups, but most capillary lumens could not be measured, and hence the inter-group variation of capillary size is unknown. The relative constancy of K^trans^/rCBV across various experimental conditions, in marked contrast to that of K^trans^, indicates constancy of P_CA_/d, for which the most parsimonius explanation is that P_CA_ and d each remain constant, rather than synchronously covarying. The analysis suggests that CBV changes, and the associated changes in total vessel surface area, are key factors driving the elevation of K^trans^ with tumor growth and/or the differences in K^trans^ between experimental groups in our study. The increasing K^trans^ that we observe with tumor growth in the untreated group for example, is more likely due to an expansion of the tumor vascular network, than a further compromising of the blood-brain barrier. Conversely, the reduction of K^trans^ in the cediranib/SC68896 group versus untreated is likely to be primarily related to the concomitant reduction in CBV and total vessel surface area. Similarly, the tendency of Cediranib to reduce K^trans^ may also primarily reflect CBV changes. A significant difference in K^trans^/rCBV was noted (versus untreated) for the SC68896 treated group but was moderate in magnitude, suggesting that increased permeability constitutes a measurable but still minor contribution to the high K^trans^ value observed in that group.

The K^trans^ value at an early stage of tumor growth (~0.1 min^−1^, Fig 3) is approximately four orders of magnitude greater that that reported for normal brain (~0.00001 min^−1^) and hence requires substantially greater vascular permeability to account for it.[68] However, with further tumor growth, K^trans^/rCBV remains relatively unchanged, and it is reasonable to conclude that in tumor vasculature there are regulated limits in P_CA_. The multi-parametric approach we employed, and analysis of the K^trans^/rCBV ratio, adds diagnostic power and could improve the utility of dynamic MRI in drug efficacy assessment, known to have limitations when restricted to considerations of K^trans^.[69] Our findings highlight the importance of correctly interpreting changes in K^trans^, which is often incorrectly considered to be equivalent to vascular permeability itself. Caution regarding K^trans^ interpretation should also extend to studies employing Evans blue dye, commonly employed as a measure of vascular permeability in tumors, but like K^trans^ is actually a measure of the extent of vascular extravasation.

Our study documented an *in vivo* synergy between cediranib and SC68896 in slowing glioma tumor growth, extending survival, inducing tumor necrosis, and remodeling the vasculature. Further studies are required to define the underlying mechanisms which drive this synergistic interaction. Our observation of anti-angiogenic synergy could be explained by the known effects of proteasome inhibition on angiogenic signaling which may effectively complement those of cediranib.[20–33] Concomitant with a vascular normalization process, improved drug delivery may then occur, resulting in increased anti-glioma efficacy. The synergy is likely to derive from more than anti-angiogenic effects alone however, as various biological effects resulting from proteasome inhibition could induce synergism with the anti-angiogenic RTK inhibitor cediranib. Proteasome inhibitors create a pro-apoptotic cellular environment, through their effects on critical apoptotic regulatory proteins. Recently, the efficacy of proteasome inhibitors has been related to cell death triggered by the unfolded protein response, which is induced by ER (endoplasmic reticulum) stress, a condition prevalent under hypoxic conditions, and thus potentially, anti-angiogenic treatment.[46, 47, 70–73] Proteasome inhibitors also inhibit the HIF1α transcription factor, which drives critical aspects of the cells’ defensive response to hypoxia.[17–19, 34–38] Possibly of importance in regards to synergistic interactions is inhibition of Akt/mTOR signaling, which also is critically involved in angiogenesis, survival and proliferation. Notably, proteasome inhibition has been shown to inhibit Akt/mTOR signaling.[34, 39–45] We have shown that cediranib also inhibits Akt in 4C8 glioma, consistent with that reported for U251 glioma.[6, 9] Recently, a study reported that sunitinib, a receptor tyrosine kinase inhibitor which like cediranib targets VEGF, PDGF and c-Kit signaling, can effectively sensitize metastatic melanoma cell cultures to bortezomib treatment and cause a synergistic reduction in cell viability through a combined inhibition of the pro-survival PI3K/Akt signaling pathway.[44] Akt inhibition was also strongly implicated in the synergistic interaction observed between bortezomib and sorafenib, a receptor tyrosine kinase inhibitor targeting Raf, VEGF and PDGF signaling, in a variety of solid tumor models *in vitro* including renal cell cancer, cervical cancer, breast cancer, hepatocellular carcinoma and glioma.[45] Notably, they also reported that the combined agents synergistically reduced phosphorylation of VEFG receptor-2 and PDGF receptor-β in chronic myelogenous leukemia cells. The potent effect on these critical angiogenic targets with combined proteasome and VEGF/PDGF RTK inhibition is consistent with the synergistic anti-angiogenic effect of cediranib/SC68896 observed in the current study. Furthermore, the markedly increased necrosis which we observed with combined cediranib/SC68896 would be consistent with an increased induction of cell death through effects on the various cell survival mechanisms discussed above. The increased regional necrosis with cediranib/SC68896 was observed in tandem with unchanged viable tissue levels of cleaved caspase-3, suggesting that the combined treatment promoted cell death heterogeneously within the tumor tissue. This is consistent with the known heterogeneity of high-grade glioma, in terms of vascularity, hypoxia, and necrosis, which is well replicated by 4C8 glioma.[1, 8, 9, 56] The mean percent tumor necrosis we observed in our study, varied between ~2–12% in the various treatment groups. In a recent study quantifying tumor characteristics in 83 glioblastoma patients, percent necrosis at the time of diagnosis was found to be highly variable, with a mean of 19% of tumor volume.[74] The somewhat lower values observed in our intracranial mouse glioma study could be related to the substantial difference in tumor volume and/or the time over which tumor growth evolved. Differences in Ki67 were not detected between experimental groups, suggesting that the synergistic cytotoxicity of cediranib/SC68896 is unrelated to a reduction in cell proliferation. The absence of a detectable difference could also be related in part to the late tumor stage at which histology was obtained, as differences in the rate of tumor growth were more obvious at earlier stages (Fig 1). While further studies are required to unravel the underlying mechanisms, our study, using a highly vascular mouse model of malignant glioma, provides the first direct *in vivo* evidence that proteasome inhibition greatly enhances the anti-tumor/anti-angiogenic effect of a VEGF/PDGF RTK inhibitor. Given the potent resistance of malignant glioma to anti-angiogenic treatment, and the documented failure of bevacizumab or small molecule RTK anti-angiogenics to extend clinical survival, our study provides compelling evidence for the potential utility of combined proteasome inhibition. [5, 11–16]

In conclusion, our results indicate that combined cediranib/SC68896 treatments synergistically induce reduced tumor growth, improved survival and increased tumor necrosis in intracranial 4C8 glioma. The combination effectively induced potent anti-angiogenic effects, reducing rCBF, rCBV, rMTT and K^trans^ in comparison to untreated, consistent with a process of vascular normalization. Our studies provide a rationale for further exploration of this unique therapeutic approach in the clinical setting for malignant glioma.

## Supporting Information

S1 Appendix Contrast Agent Capillary Extravasation. Derivation of relationship between CBV and permeability measures P_CA_ and k_pe._
(DOCX)

## References

[1] Gladson CL, Prayson RA, Liu WM. The pathobiology of glioma tumors. Annu Rev Pathol. 2010;5:33–50. 10.1146/annurev-pathol-121808-102109 19737106 PMC2887670

[2] Stupp R, Mason WP, van den Bent MJ, Weller M, Fisher B, Taphoorn MJ, et al Radiotherapy plus concomitant and adjuvant temozolomide for glioblastoma. N Engl J Med. 2005;352(10):987–96. 15758009 10.1056/NEJMoa043330

[3] Van Meir EG, Hadjipanayis CG, Norden AD, Shu HK, Wen PY, Olson JJ. Exciting new advances in neuro-oncology: the avenue to a cure for malignant glioma. CA Cancer J Clin. 2010;60(3):166–93. 10.3322/caac.20069 20445000 PMC2888474

[4] Thaker NG, Pollack IF. Molecularly targeted therapies for malignant glioma: rationale for combinatorial strategies. Expert Rev Neurother. 2009;9(12):1815–36. 10.1586/ern.09.116 19951140 PMC2819818

[5] Bergers G, Hanahan D. Modes of resistance to anti-angiogenic therapy. Nat Rev Cancer. 2008;8(8):592–603. 10.1038/nrc2442 18650835 PMC2874834

[6] Martinho O, Silva-Oliveira R, Miranda-Goncalves V, Clara C, Almeida JR, Carvalho AL, et al In Vitro and In Vivo Analysis of RTK Inhibitor Efficacy and Identification of Its Novel Targets in Glioblastomas. Transl Oncol. 2013;6(2):187–96. 23544171 10.1593/tlo.12400 PMC3610556

[7] Wedge SR, Kendrew J, Hennequin LF, Valentine PJ, Barry ST, Brave SR, et al AZD2171: a highly potent, orally bioavailable, vascular endothelial growth factor receptor-2 tyrosine kinase inhibitor for the treatment of cancer. Cancer Res. 2005;65(10):4389–400. 15899831 10.1158/0008-5472.CAN-04-4409

[8] Lobo MR, Green SC, Schabel MC, Gillespie GY, Woltjer RL, Pike MM. Quinacrine synergistically enhances the antivascular and antitumor efficacy of cediranib in intracranial mouse glioma. Neuro Oncol. 2013;15(12):1673–83. 10.1093/neuonc/not119 24092859 PMC3829589

[9] Lobo MR, Wang X, Gillespie GY, Woltjer RL, Pike MM. Combined Efficacy of Cediranib and Quinacrine in Glioma Is Enhanced by Hypoxia and Causally Linked to Autophagic Vacuole Accumulation. PLoS One. 2014;9(12):e114110 10.1371/journal.pone.0114110 25490024 PMC4260788

[10] Kamoun WS, Ley CD, Farrar CT, Duyverman AM, Lahdenranta J, Lacorre DA, et al Edema control by cediranib, a vascular endothelial growth factor receptor-targeted kinase inhibitor, prolongs survival despite persistent brain tumor growth in mice. J Clin Oncol. 2009;27(15):2542–52. 10.1200/JCO.2008.19.9356 19332720 PMC2739611

[11] Tabatabai G, Stupp R. Primetime for antiangiogenic therapy. Curr Opin Neurol. 2009;22(6):639–44. 10.1097/WCO.0b013e328332ba28 19786873

[12] Rahman R, Smith S, Rahman C, Grundy R. Antiangiogenic therapy and mechanisms of tumor resistance in malignant glioma. J Oncol. 2010;2010:251231 10.1155/2010/251231 20414333 PMC2855058

[13] Gotink KJ, Verheul HM. Anti-angiogenic tyrosine kinase inhibitors: what is their mechanism of action? Angiogenesis. 2010;13(1):1–14. 10.1007/s10456-009-9160-6 20012482 PMC2845892

[14] Norden AD, Drappatz J, Wen PY. Antiangiogenic therapies for high-grade glioma. Nat Rev Neurol. 2009;5(11):610–20. 10.1038/nrneurol.2009.159 19826401

[15] Hardee ME, Zagzag D. Mechanisms of glioma-associated neovascularization. Am J Pathol. 2012;181(4):1126–41. 10.1016/j.ajpath.2012.06.030 22858156 PMC3463636

[16] Phillips HS, Sandmann T, Li C, Cloughesy TF, Chinot OL, Wick W. Correlation of molecular subtypes with survival in AVAglio (bevacizumab [Bv] and radiotherapy [RT] and temozolomide [T] for newly diagnosed glioblastoma [GB]). J Clin Oncol 2014;32 ((15 Suppl)): abstract 2001.

[17] Kaur B, Khwaja FW, Severson EA, Matheny SL, Brat DJ, Van Meir EG. Hypoxia and the hypoxia-inducible-factor pathway in glioma growth and angiogenesis. Neuro Oncol. 2005;7(2):134–53. 15831232 10.1215/S1152851704001115 PMC1871894

[18] Semenza GL. Targeting HIF-1 for cancer therapy. Nat Rev Cancer. 2003;3(10):721–32. 13130303 10.1038/nrc1187

[19] Zhang H, Bosch-Marce M, Shimoda LA, Tan YS, Baek JH, Wesley JB, et al Mitochondrial autophagy is an HIF-1-dependent adaptive metabolic response to hypoxia. J Biol Chem. 2008;283(16):10892–903. 10.1074/jbc.M800102200 18281291 PMC2447655

[20] Belloni D, Veschini L, Foglieni C, Dell'Antonio G, Caligaris-Cappio F, Ferrarini M, et al Bortezomib induces autophagic death in proliferating human endothelial cells. Exp Cell Res. 2009;316(6):1010–8. 10.1016/j.yexcr.2009.11.005 19917281

[21] Boccadoro M, Morgan G, Cavenagh J. Preclinical evaluation of the proteasome inhibitor bortezomib in cancer therapy. Cancer Cell Int. 2005;5(1):18 15929791 10.1186/1475-2867-5-18 PMC1164423

[22] Brignole C, Marimpietri D, Pastorino F, Nico B, Di Paolo D, Cioni M, et al Effect of bortezomib on human neuroblastoma cell growth, apoptosis, and angiogenesis. J Natl Cancer Inst. 2006;98(16):1142–57. 16912267 10.1093/jnci/djj309

[23] Daniel KG, Kuhn DJ, Kazi A, Dou QP. Anti-angiogenic and anti-tumor properties of proteasome inhibitors. Curr Cancer Drug Targets. 2005;5(7):529–41. 16305349 10.2174/156800905774574075

[24] Drexler HC, Risau W, Konerding MA. Inhibition of proteasome function induces programmed cell death in proliferating endothelial cells. FASEB J. 2000;14(1):65–77. 10627281 10.1096/fasebj.14.1.65

[25] LeBlanc R, Catley LP, Hideshima T, Lentzsch S, Mitsiades CS, Mitsiades N, et al Proteasome inhibitor PS-341 inhibits human myeloma cell growth in vivo and prolongs survival in a murine model. Cancer Res. 2002;62(17):4996–5000. 12208752

[26] Matsuo Y, Sawai H, Ochi N, Yasuda A, Sakamoto M, Takahashi H, et al Proteasome inhibitor MG132 inhibits angiogenesis in pancreatic cancer by blocking NF-kappaB activity. Dig Dis Sci. 2009;55(4):1167–76. 10.1007/s10620-009-0814-4 19399612

[27] Rahimi N. The ubiquitin-proteasome system meets angiogenesis. Mol Cancer Ther. 2012;11(3):538–48. 10.1158/1535-7163.MCT-11-0555 22357635 PMC3297735

[28] Roccaro AM, Hideshima T, Raje N, Kumar S, Ishitsuka K, Yasui H, et al Bortezomib mediates antiangiogenesis in multiple myeloma via direct and indirect effects on endothelial cells. Cancer Res. 2006;66(1):184–91. 16397231 10.1158/0008-5472.CAN-05-1195

[29] Sunwoo JB, Chen Z, Dong G, Yeh N, Crowl Bancroft C, Sausville E, et al Novel proteasome inhibitor PS-341 inhibits activation of nuclear factor-kappa B, cell survival, tumor growth, and angiogenesis in squamous cell carcinoma. Clin Cancer Res. 2001;7(5):1419–28. 11350913

[30] Veschini L, Belloni D, Foglieni C, Cangi MG, Ferrarini M, Caligaris-Cappio F, et al Hypoxia-inducible transcription factor-1 alpha determines sensitivity of endothelial cells to the proteosome inhibitor bortezomib. Blood. 2007;109(6):2565–70. 17110461 10.1182/blood-2006-06-032664

[31] Jiang L, Song L, Wu J, Yang Y, Zhu X, Hu B, et al Bmi-1 promotes glioma angiogenesis by activating NF-kappaB signaling. PLoS One. 2013;8(1):e55527 10.1371/journal.pone.0055527 23383216 PMC3561301

[32] Peng L, Liu A, Shen Y, Xu HZ, Yang SZ, Ying XZ, et al Antitumor and anti-angiogenesis effects of thymoquinone on osteosarcoma through the NF-kappaB pathway. Oncol Rep. 2013;29(2):571–8. 10.3892/or.2012.2165 23232982

[33] Xie TX, Xia Z, Zhang N, Gong W, Huang S. Constitutive NF-kappaB activity regulates the expression of VEGF and IL-8 and tumor angiogenesis of human glioblastoma. Oncol Rep. 2010;23(3):725–32. 20127012

[34] Befani CD, Vlachostergios PJ, Hatzidaki E, Patrikidou A, Bonanou S, Simos G, et al Bortezomib represses HIF-1alpha protein expression and nuclear accumulation by inhibiting both PI3K/Akt/TOR and MAPK pathways in prostate cancer cells. J Mol Med (Berl). 2012;90(1):45–54. 21909688 10.1007/s00109-011-0805-8

[35] Birle DC, Hedley DW. Suppression of the hypoxia-inducible factor-1 response in cervical carcinoma xenografts by proteasome inhibitors. Cancer Res. 2007;67(4):1735–43. 17308115 10.1158/0008-5472.CAN-06-2722

[36] Kaluz S, Kaluzova M, Stanbridge EJ. Does inhibition of degradation of hypoxia-inducible factor (HIF) alpha always lead to activation of HIF? Lessons learnt from the effect of proteasomal inhibition on HIF activity. J Cell Biochem. 2008;104(2):536–44. 18059036 10.1002/jcb.21644

[37] Shin DH, Chun YS, Lee DS, Huang LE, Park JW. Bortezomib inhibits tumor adaptation to hypoxia by stimulating the FIH-mediated repression of hypoxia-inducible factor-1. Blood. 2008;111(6):3131–6. 10.1182/blood-2007-11-120576 18174379

[38] Shin DH, Li SH, Chun YS, Huang LE, Kim MS, Park JW. CITED2 mediates the paradoxical responses of HIF-1alpha to proteasome inhibition. Oncogene. 2008;27(13):1939–44. 17906695 10.1038/sj.onc.1210826

[39] Chen KF, Yeh PY, Hsu C, Hsu CH, Lu YS, Hsieh HP, et al Bortezomib overcomes tumor necrosis factor-related apoptosis-inducing ligand resistance in hepatocellular carcinoma cells in part through the inhibition of the phosphatidylinositol 3-kinase/Akt pathway. J Biol Chem. 2009;284(17):11121–33. 10.1074/jbc.M806268200 19261616 PMC2670117

[40] Chen KF, Yeh PY, Yeh KH, Lu YS, Huang SY, Cheng AL. Down-regulation of phospho-Akt is a major molecular determinant of bortezomib-induced apoptosis in hepatocellular carcinoma cells. Cancer Res. 2008;68(16):6698–707. 10.1158/0008-5472.CAN-08-0257 18701494

[41] Inoue T, Shiraki K, Fuke H, Yamanaka Y, Miyashita K, Yamaguchi Y, et al Proteasome inhibition sensitizes hepatocellular carcinoma cells to TRAIL by suppressing caspase inhibitors and AKT pathway. Anticancer Drugs. 2006;17(3):261–8. 16520654 10.1097/00001813-200603000-00004

[42] Khan T, Stauffer JK, Williams R, Hixon JA, Salcedo R, Lincoln E, et al Proteasome inhibition to maximize the apoptotic potential of cytokine therapy for murine neuroblastoma tumors. J Immunol. 2006;176(10):6302–12. 16670342 10.4049/jimmunol.176.10.6302

[43] Noguchi M, Hirata N, Suizu F. The links between AKT and two intracellular proteolytic cascades: Ubiquitination and autophagy. Biochim Biophys Acta. 2014;1846(2):342–52. 10.1016/j.bbcan.2014.07.013 25109892

[44] Yeramian A, Sorolla A, Velasco A, Santacana M, Dolcet X, Valls J, et al Inhibition of activated receptor tyrosine kinases by Sunitinib induces growth arrest and sensitizes melanoma cells to Bortezomib by blocking Akt pathway. Int J Cancer. 2012;130(4):967–78. 10.1002/ijc.26096 21445974

[45] Yu C, Friday BB, Lai JP, Yang L, Sarkaria J, Kay NE, et al Cytotoxic synergy between the multikinase inhibitor sorafenib and the proteasome inhibitor bortezomib in vitro: induction of apoptosis through Akt and c-Jun NH2-terminal kinase pathways. Mol Cancer Ther. 2006;5(9):2378–87. 16985072 10.1158/1535-7163.MCT-06-0235

[46] Almond JB, Cohen GM. The proteasome: a novel target for cancer chemotherapy. Leukemia. 2002;16(4):433–43. 11960320 10.1038/sj.leu.2402417

[47] McConkey DJ, Zhu K. Mechanisms of proteasome inhibitor action and resistance in cancer. Drug Resist Updat. 2008;11(4–5):164–79. 10.1016/j.drup.2008.08.002 18818117

[48] Milano A, Perri F, Caponigro F. The ubiquitin-proteasome system as a molecular target in solid tumors: an update on bortezomib. Onco Targets Ther. 2009;2:171–8. 20616904 10.2147/ott.s4503 PMC2886336

[49] Roth P, Kissel M, Herrmann C, Eisele G, Leban J, Weller M, et al SC68896, a novel small molecule proteasome inhibitor, exerts antiglioma activity in vitro and in vivo. Clin Cancer Res. 2009;15(21):6609–18. 10.1158/1078-0432.CCR-09-0548 19825946

[50] Sterz J, von Metzler I, Hahne JC, Lamottke B, Rademacher J, Heider U, et al The potential of proteasome inhibitors in cancer therapy. Expert Opin Investig Drugs. 2008;17(6):879–95. 10.1517/13543784.17.6.879 18491989

[51] Buac D, Shen M, Schmitt S, Kona FR, Deshmukh R, Zhang Z, et al From bortezomib to other inhibitors of the proteasome and beyond. Curr Pharm Des. 2013;19(22):4025–38. 23181572 10.2174/1381612811319220012 PMC3657018

[52] Driscoll JJ, Woodle ES. Targeting the ubiquitin+proteasome system in solid tumors. Semin Hematol. 2012;49(3):277–83. 10.1053/j.seminhematol.2012.04.002 22726552

[53] Labussiere M, Pinel S, Delfortrie S, Plenat F, Chastagner P. Proteasome inhibition by bortezomib does not translate into efficacy on two malignant glioma xenografts. Oncol Rep. 2008;20(5):1283–7. 18949434

[54] de Wilt LH, Kroon J, Jansen G, de Jong S, Peters GJ, Kruyt FA. Bortezomib and TRAIL: a perfect match for apoptotic elimination of tumour cells? Crit Rev Oncol Hematol. 2013;85(3):363–72. 10.1016/j.critrevonc.2012.08.001 22944363

[55] Dyer CA, Philibotte T. A clone of the MOCH-1 glial tumor in culture: multiple phenotypes expressed under different environmental conditions. J Neuropathol Exp Neurol. 1995;54(6):852–63. 7595658 10.1097/00005072-199511000-00012

[56] Pike MM, Stoops CN, Langford CP, Akella NS, Nabors LB, Gillespie GY. High-resolution longitudinal assessment of flow and permeability in mouse glioma vasculature: Sequential small molecule and SPIO dynamic contrast agent MRI. Magn Reson Med. 2009;61(3):615–25. 10.1002/mrm.21931 19235262 PMC3243360

[57] Sourbron SP, Buckley DL. Tracer kinetic modelling in MRI: estimating perfusion and capillary permeability. Phys Med Biol. 2012;57(2):R1–33. 10.1088/0031-9155/57/2/R1 22173205

[58] Tofts PS, Brix G, Buckley DL, Evelhoch JL, Henderson E, Knopp MV, et al Estimating kinetic parameters from dynamic contrast-enhanced T(1)-weighted MRI of a diffusable tracer: standardized quantities and symbols. J Magn Reson Imaging. 1999;10(3):223–32. 10508281 10.1002/(sici)1522-2586(199909)10:3<223::aid-jmri2>3.0.co;2-s

[59] Schabel MC, DiBella EV, Jensen RL, Salzman KL. A model-constrained Monte Carlo method for blind arterial input function estimation in dynamic contrast-enhanced MRI: II. In vivo results. Phys Med Biol. 2010;55(16):4807–23. 10.1088/0031-9155/55/16/012 20679695 PMC3533373

[60] Schabel MC, Fluckiger JU, DiBella EV. A model-constrained Monte Carlo method for blind arterial input function estimation in dynamic contrast-enhanced MRI: I. Simulations. Phys Med Biol. 2010;55(16):4783–806. 10.1088/0031-9155/55/16/011 20679691 PMC3533367

[61] Ostergaard L, Weisskoff R, Chesler D, Gyldensted C. High resolution measurement of cerebral blood flow using intravascular tracer bolus passages. Part I: Mathematical approach and statistical analysis. MRM. 1996;36:715–25. 8916022 10.1002/mrm.1910360510

[62] Tuominen VJ, Ruotoistenmaki S, Viitanen A, Jumppanen M, Isola J. ImmunoRatio: a publicly available web application for quantitative image analysis of estrogen receptor (ER), progesterone receptor (PR), and Ki-67. Breast Cancer Res. 2010;12(4):R56 10.1186/bcr2615 20663194 PMC2949645

[63] Leban J, Blisse M, Krauss B, Rath S, Baumgartner R, Seifert MH. Proteasome inhibition by peptide-semicarbazones. Bioorg Med Chem. 2008;16(8):4579–88. 10.1016/j.bmc.2008.02.042 18313310

[64] Goel S, Wong AH, Jain RK. Vascular normalization as a therapeutic strategy for malignant and nonmalignant disease. Cold Spring Harb Perspect Med. 2012;2(3):a006486 10.1101/cshperspect.a006486 22393532 PMC3282493

[65] Winkler F, Kozin SV, Tong RT, Chae SS, Booth MF, Garkavtsev I, et al Kinetics of vascular normalization by VEGFR2 blockade governs brain tumor response to radiation: role of oxygenation, angiopoietin-1, and matrix metalloproteinases. Cancer Cell. 2004;6(6):553–63. 15607960 10.1016/j.ccr.2004.10.011

[66] Sorensen AG, Emblem KE, Polaskova P, Jennings D, Kim H, Ancukiewicz M, et al Increased survival of glioblastoma patients who respond to antiangiogenic therapy with elevated blood perfusion. Cancer Res. 2012;72(2):402–7. 10.1158/0008-5472.CAN-11-2464 22127927 PMC3261301

[67] Li X, Springer CS Jr, Jerosch-Herold M. First-pass dynamic contrast-enhanced MRI with extravasating contrast reagent: evidence for human myocardial capillary recruitment in adenosine-induced hyperemia. NMR Biomed. 2009;22(2):148–57. 10.1002/nbm.1293 18727151

[68] Njus JM, Li X, Springer CS, Taylor M, Greisel T, Telang FW, et al Changes in blood-brain barrier permeability and blood volume during MS lesion development and evolution. Proc Int Soc Magn Reson Med. 2008;16(3431).

[69] O'Connor JP, Jackson A, Parker GJ, Roberts C, Jayson GC. Dynamic contrast-enhanced MRI in clinical trials of antivascular therapies. Nat Rev Clin Oncol. 2012;9(3):167–77. 10.1038/nrclinonc.2012.2 22330689

[70] Fels DR, Ye J, Segan AT, Kridel SJ, Spiotto M, Olson M, et al Preferential cytotoxicity of bortezomib toward hypoxic tumor cells via overactivation of endoplasmic reticulum stress pathways. Cancer Res. 2008;68(22):9323–30. 10.1158/0008-5472.CAN-08-2873 19010906 PMC3617567

[71] Healy SJ, Gorman AM, Mousavi-Shafaei P, Gupta S, Samali A. Targeting the endoplasmic reticulum-stress response as an anticancer strategy. Eur J Pharmacol. 2009;625(1–3):234–46. 10.1016/j.ejphar.2009.06.064 19835867

[72] Kardosh A, Golden EB, Pyrko P, Uddin J, Hofman FM, Chen TC, et al Aggravated endoplasmic reticulum stress as a basis for enhanced glioblastoma cell killing by bortezomib in combination with celecoxib or its non-coxib analogue, 2,5-dimethyl-celecoxib. Cancer Res. 2008;68(3):843–51. 10.1158/0008-5472.CAN-07-5555 18245486

[73] Nawrocki ST, Carew JS, Pino MS, Highshaw RA, Dunner K Jr, Huang P, et al Bortezomib sensitizes pancreatic cancer cells to endoplasmic reticulum stress-mediated apoptosis. Cancer Res. 2005;65(24):11658–66. 16357177 10.1158/0008-5472.CAN-05-2370

[74] Nestler U, Lutz K, Pichlmeier U, Stummer W, Franz K, Reulen HJ, et al Anatomic features of glioblastoma and their potential impact on survival. Acta Neurochir (Wien). 2015;157(2):179–86. 25391974 10.1007/s00701-014-2271-x

