# Supplementary material for: Synergistic Antivascular and Antitumor Efficacy with Combined Cediranib and SC6889 in Intracranial Mouse Glioma
Source: PLoS One. 2015 Dec 8;10(12):e0144488. doi: 10.1371/journal.pone.0144488 (PMC4672903; doi:10.1371/journal.pone.0144488)
Supplement: S1 Appendix — Derivation of relationship between CBV and permeability measures PCA and kpe. (DOCX) [file pone.0144488.s001.docx]

**S1 Appendix. Contrast Agent Capillary Extravasation**. Derivation of relationship between CBV and permeability measures P_CA_ and k_pe._

The vast majority of contrast agent (CA) extravasation occurs from capillary microvessels. We treat these as right cylinders. Thus, the surface area, A, is given in Equation [A1], where: d is the diameter, and L the length. The volume, V, is given

A = πdL [A1]

in Equation [A2]. Thus, A is related to V as in Equation [A3]. It is important to note that A, d,

V = πd^2^L/4 [A2]

A = (4/d)V [A3]

and V represent the *average,* *individual* capillary quantities in a tissue voxel or region-of-interest [ROI].

The transfer constant for contrast agent (CA) capillary extravasation is K^trans^. The CBF‑dependence of CA extravasation is given by the Renkin-Crone equation (Li, *et al*, 2009).[67]. However, for Gd chelate CAs in most tissues, the permeation-limited Renkin-Crone expression pertains (Li, *et al*, 2009) [67], and that is given in Equation [A4], where: P_CA_ is the *average* vascular wall CA permeability coefficient, and S is

K^trans^ ≅ P_CA_S [A4]

the *total* vascular surface area per tissue voxel or ROI volume. However, S can be further expressed in Equation [A5], where: ρ^†^ is the capillary number density [the number (n)

S = (n/V_T_)A = ρ^†^A [A5]

of capillaries per tissue voxel or ROI volume (V_T_)]. Combining Eqs. [A5] with [A3] gives Equation [A6]. At the same time, the *total* blood volume per tissue voxel or ROI volume,

S = ρ^†^(4/d)V [A6]

v_b_, [the blood volume fraction] is given in Equation [A7] (Rooney, et al, 2015).[74] Eliminating

v_b_ = ρ^†^V [A7]

ρ^†^V from Eqs. [A6] and [A7] yields Equation [A8]. Rearranging gives Equation [A9].

S = 4 (v_b_)/d [A8]

v_b_ = dS/4 [A9]

The CBV measured by DSC-MRI here is **∝** v_b_. Thus, we can take the K^trans^ to CBV ratio and see in Equation [A10] that K^trans^/CBV is proportional to the P_CA_/d ratio.

K^trans^/CBV **∝** 4(P_CA_/d) [A10]

But, 4(P_CA_/d) = k_pe_, the unidirectional rate constant for CA extravasation (Rooney, et al, 2015).[1] Thus, K^trans^/CBV is proportional to k_pe_, Equation [A11].

K^trans^/CBV **∝** k_pe_ [A11]

**References**

1. Rooney WD, Li X, Sammi MK, Bourdette DN, Neuwelt EA, Springer CS, Jr. Mapping human brain capillary water lifetime: high-resolution metabolic neuroimaging. NMR Biomed. 2015;28(6):607-23.
